# Short-term role of the dietary total antioxidant capacity in two hypocaloric regimes on obese with metabolic syndrome symptoms: the RESMENA randomized controlled trial

**DOI:** 10.1186/1743-7075-10-22

**Published:** 2013-02-13

**Authors:** Patricia Lopez-Legarrea, Rocio de la Iglesia, Itziar Abete, Isabel Bondia-Pons, Santiago Navas-Carretero, Lluis Forga, J Alfredo Martinez, M Angeles Zulet

**Affiliations:** 1 Department of Nutrition, Food Science and Physiology, University of Navarra, Irunlarrea rd 1, Pamplona, 31008, Spain; 2 Biodonostia Health Research Institute, Doctor Begiristain rd (no number), San Sebastian, 20014, Spain; 3 Hospital Complex of Navarra, Irunlarrea rd 3, Pamplona, 31008, Spain; 4 CIBERobn. Carlos III Health Research Institute, Madrid, Spain

**Keywords:** Antioxidant, Weight loss, Energy restriction, Macronutrient distribution, Dietary components, Nutritional profile

## Abstract

**Background:**

Dietary strategies seem to be the most prescribed therapy in order to counteract obesity regarding not only calorie restriction, but also bioactive ingredients and the composition of the consumed foods. Dietary total antioxidant capacity (TAC) is gaining importance in order to assess the quality of the diet.

**Methods:**

Ninety-six obese adults presenting metabolic syndrome (MetS) symptoms completed an 8-week intervention trial to evaluate the effects of a novel dietary program with changes in the nutrient distribution and meal frequency and to compare it with a dietary pattern based on the American Heart Association (AHA) guidelines.

Anthropometric and biochemical parameters were assessed at baseline and at the endpoint of the study, in addition to 48-hours food dietary records.

**Results:**

Both diets equally (*p* > 0.05) improved MetS manifestations. Dietary TAC was the component which showed the major influence on body weight (*p* = 0.034), body mass index (*p* = 0.026), waist circumference (*p* = 0.083) and fat mass (*p* = 0.015) reductions. Transaminases (ALT and AST) levels (*p* = 0.062 and *p* = 0.004, respectively) were associated with lower TAC values.

**Conclusion:**

RESMENA diet was as effective as AHA pattern for reducing MetS features. Dietary TAC was the most contributing factor involved in body weight and obesity related markers reduction.

**Trial registration:**

http://www.clinicaltrials.gov; NCT01087086

## Background

The World Health Organization (WHO) estimates that at least 300 million people are obese nowadays [1]. Obesity, is strongly associated with comorbidities such as impaired glucose tolerance or diabetes, insulin resistance, dyslipidemia, hypertension, nonalcoholic fatty liver disease, hyperuricemia, and prothrombotic and proinflammatory states, which are related to the onset of metabolic syndrome (MetS) [2-4]. Also according to the WHO estimations, obesity prevalence rates will tend to increase in the next years. So that new effective proposals are needed in order to prevent/counteract the obesity onset and spread.

Dietary strategies are one of the most prescribed therapies to prevent/counteract overweight and obesity [5]. While dietetic programs have traditionally focused on calorie restriction, new nutritional alternatives are nowadays being investigated. They entail macronutrient distribution [6], meal frequency [7], consumption of bioactive ingredients, such as fiber [8] and n-3 fatty acids [9], glycemic index (GI)/glycemic load (GL) [10] or the dietary total antioxidant capacity (TAC) [11]. Dietary TAC is considered an appropriate approach to measure the cumulative antioxidant properties of food [12], despite its controversial use at evaluating the role of antioxidants *in vivo*[13]. Oxidative stress is suggested to be involved in the onset of several obesity-related disorders such as hypertension, dyslipidemia, type-2 Diabetes Mellitus and MetS [11]. In this context, dietary TAC is gaining importance as a valuable tool to investigate the relationship between diet and oxidative stress-related diseases [14]. Furthermore, the influence of the dietary TAC has been poorly investigated in the context of MetS.

Many studies have separately examined the impact of different dietary components, such as macronutrient distribution [15], meal frequency [7], fiber [8], n-3 fatty acids [16], GI/GL [17] or dietary TAC [18]. However, to date, they have not been integrated together on a dietetic plan based on habitual foods intake to combat excessive fat deposition. In this context, the RESMENA-S (MEtabolic Syndrome REduction in NAvarra-Spain) study (http://www.clinicaltrials.gov; NCT01087086) [19] aimed at evaluating the effect of a novel dietary strategy involving a modified macronutrient distribution, higher meal frequency, increased fiber and n-3 fatty acids consumption, low GI/GL and high TAC food and at comparing it with the American Heart Association (AHA) guidelines, which is currently considered as a reference dietary pattern to reduce fat mass content and improve MetS markers [20].

## Methods

### Study population

**Figure 1:**
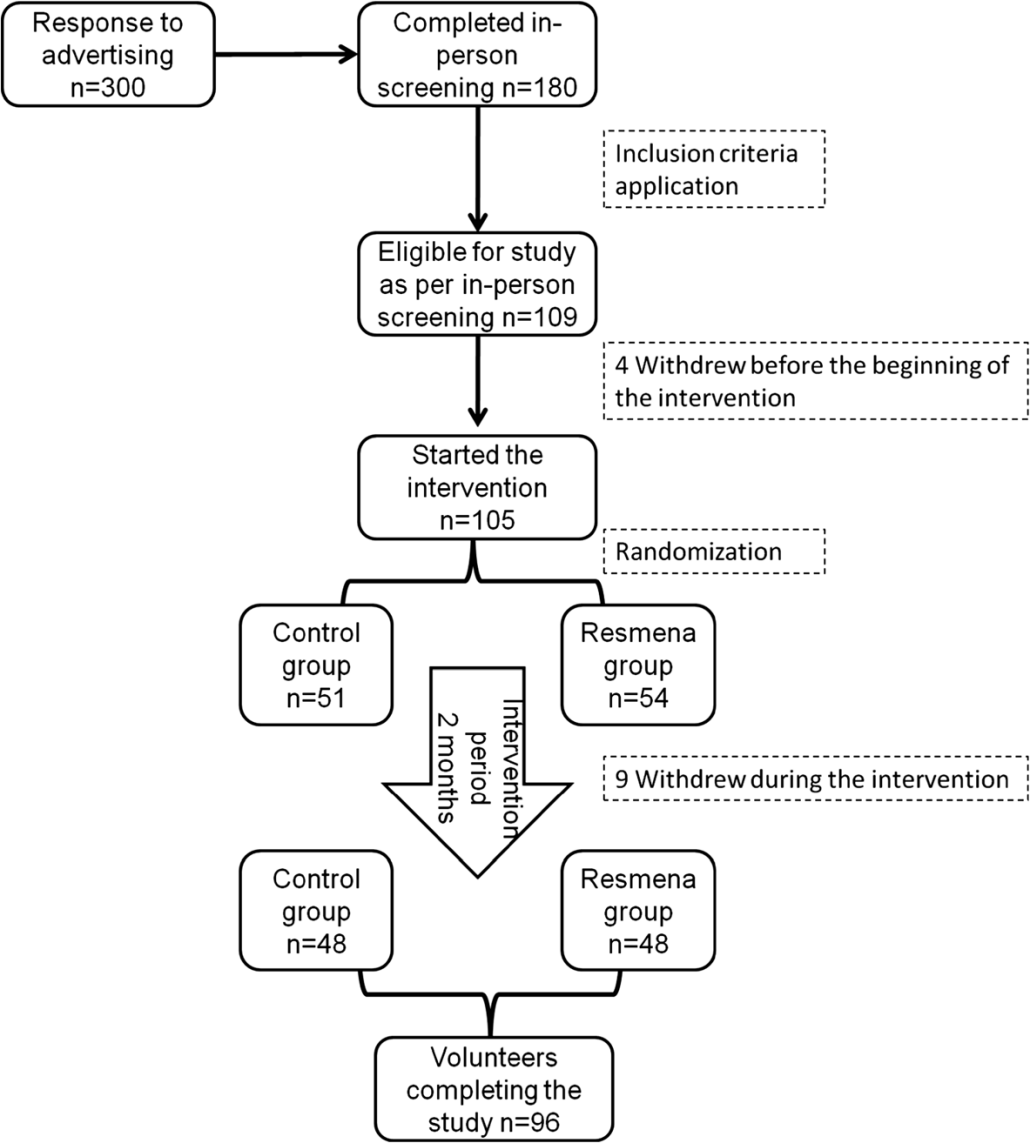
Flowchart of the study subjects from advertisement through to the end of the 8-week intervention.

One hundred and five (56 Male and 49 Female) caucasian adults (49 ± 10 years old) presenting obesity determined by a Body Mass Index (BMI) higher than 30 Kg/m^2^ (mean BMI = 35.85 ± 4.67 kg/m^2^) and at least two MetS signs according to the International Diabetes Federation criteria [21] were enrolled in the study and 96 of them completed the trial (Figure 1). The presence of psychiatric disturbances, eating disorders, chronic diseases related with the metabolism of nutrients, major body weight changes in the last three months and difficulties in changing food habits were considered as exclusion criteria. Subjects were recruited through local newspaper advertisements and the Department database. All subjects gave written informed consent (http://www.clinicaltrials.gov; NCT01087086) as approved by the Ethics Committee of the University of Navarra (065/2009) and in accordance with the Declaration of Helsinki. There were 9 dropouts along the study period. Baseline characteristics are presented in Table 1.

**Table 1:** Selected anthropometric characteristics of the whole sample and categorized by gender at baseline

**Variable**	**Total (n = 96)**	**Male**	**Female**	**p**
Sex	-	51	45	-
Age (years)	49 ± 10	48 ± 9	50 ± 10	0.194
Weight (kg)	99.73 ± 17.85	108.28 ± 15.94	90.03 ± 14.76	**<0.001**
Height (m)	1.67 ± 0.11	1.74 ± 0.08	1.58 ± 0.07	**<0.001**
BMI (kg/m^2^)	35.84 ± 4.67	35.75 ± 4.38	35.96 ± 5.02	0.822
Waist circumference (cm)	111.10 ± 12.80	116.27 ± 10.04	105.24 ± 13.16	**<0.001**
Waist/Hip ratio	0.96 ± 0.10	1.03 ± 0.07	0.89 ± 0.08	**<0.001**

*BMI*, Body Mass Index.

For the sex variable it is reported the frequency of men and females. Mean and standard deviation data are shown concerning the remaining variables. *p*-value: Comparison between men and women baseline characteristics.

*p* < 0.05 was set-up as statistically significant.

### Study protocol

A randomized, controlled trial was designed to compare the effect of two dietary strategies for weight loss with different macronutrient distribution on anthropometric measurements and biochemical markers in obese subjects with MetS manifestations. Participants were randomly assigned to the control or the experimental diet (Figure 1). The study was of six months duration in two sequential periods: one intervention period of 8 weeks in which subjects received nutritional assessment every 15 days followed by a self-control period of 4 months in which subjects followed the first period learned-habits. This work reports on the 8-weeks findings.

At each visit, anthropometric assessments and body composition by bioimpedance were measured. Fasting blood and 24-h urine samples were collected and body composition by Dual-energy X-ray Absorptiometry (DXA) was measured at baseline and at the endpoint of the intervention period.

### Diets

Two energy-restricted diets were prescribed and compared. An energy restriction of 30% was applied to the total energy requirements of each patient. Resting metabolic rate was calculated using the Harris-Benedict equation where the Wilkens adjusted weight was applied. Then, physical activity factor was considered in order to calculate total energy requirements according to the “Food and Nutrition Board, National Research Council: Recommended Dietary Allowances: 10th ed.” [22]. The Control diet was based on the AHA guidelines [20], including 3–5 meals/day, a macronutrient distribution of 50–55% total caloric value from carbohydrates, 15% from proteins and 30% from lipids, a healthy fatty acids profile, an intake of fiber of 20–25 g/day and a cholesterol recommendation of < 300 mg/day (Table 2). The RESMENA diet was composed of 7 meals/day including breakfast, lunch, dinner, two snacks in the morning and other two in the afternoon. The macronutrient distribution was as following: 40% total caloric value from carbohydrates, 30% from proteins and 30% from lipids. This pattern also maintained a healthy fatty acids profile, an input of fiber of 20–25 g/day and a cholesterol content of < 300 mg/day. It included an increased input of n-3 fatty acids, an increased amount of natural antioxidants and focused on low GI/GL carbohydrates (Table 2).

**Table 2:** Comparisons of the habitual intake, the scheduled diets, the final intake and the adherence

**Variable**	**Control group (n = 48)**			**RESMENA group (n = 48)**					
	**Habitual intake (day 0)**	**Scheduled diet**	**Final intake (day 60)**	**Habitual intake (day 0)**	**p**^**a**^	**Scheduled diet**	**p**^**b**^	**Final intake (day 60)**	**p**^**c**^
Energy (kcal/day)	2103 ± 451	1412 ± 177	1352 ± 284	2277 ± 566	0.099	1395 ± 188	0.649	1337 ± 289	0.808
CHO (g/day)	186.74 ± 58.90 (35.52%)	178.58 ± 20.15 (50.59%)	132.37 ± 35.33^*$$$*^ (39.16%)	201.66 ± 65.30 (35.43%)	0.243	128.65 ± 15.97 (36.89%)	**<0.001**	114.55 ± 31.10^*$$*^ (34.26%)	**0.013**
Protein (g/day)	93.58 ± 21.63 (17.80%)	57.01 ± 5.78 (16.14%)	60.83 ± 17.16 (18.00%)	95.01 ± 20.06 (16.69%)	0.738	99.54 ± 13.43 (28.54%)	**<0.001**	78.20 ± 17.46^*$$$*^ (23.39%)	**<0.001**
PQ	0.30 ± 0.05	0.31 ± 0.01	0.30 ± 0.05^$^	0.30 ± 0.05	0.594	0.34 ± 0.01	**<0.001**	0.28 ± 0.05^$$$^	0.070
Protein/CHO	0.54 ± 0.18	0.32 ± 0.01	0.48 ± 0.18^$$$^	0.50 ± 0.13	0.224	0.77 ± 0.01	**<0.001**	0.70 ± 0.15^$$^	**<0.001**
Total fats (g/day)	97.29 ± 27.00 (41.64%)	46.02 ± 8.69 (29.33%)	59.09 ± 15.51^*$$$*^ (39.33%)	110.26 ± 31.84 (43.58%)	**0.034**	46.98 ± 7.07 (30.31%)	0.554	56.58 ± 17.28^*$$$*^ (38.08%)	0.497
SFA (g/day)	28.89 ± 10.13 (12.36%)	14.02 ± 2.44 (8.94%)	14.48 ± 5.47 (9.64%)	34.85 ± 13.64 (13.76%)	**0.017**	12.70 ± 1.86 (8.19%)	**0.004**	16.08 ± 5.80^*$$$*^(10.82%)	0.181
MUFA (g/day)	47.35 ± 12.76 (20.26%)	20.59 ± 4.51 (13.12%)	32.25 ± 9.18^*$$$*^ (21.47%)	51.90 ± 15.42 (20.52%)	0.118	18.96 ± 3.11 (12.23%)	**0.042**	24.65 ± 7.80^*$$$*^ (16.59%)	**<0.001**
PUFA (g/day)	12.58 ± 3.80 (5.38%)	7.54 ± 1.20 (4.81%)	7.65 ± 2.87 (5.09%)	13.92 ± 3.79 (5.50%)	0.088	10.77 ± 1.31 (6.95%)	**<0.001**	10.91 ± 5.56 (7.34%)	**0.001**
n3-FA	0.46 ± 0.54	0.20 ± 0.05	0.20 ± 0.44	0.28 ± 0.43	0.079	0.83 ± 0.30	**<0.001**	0.37 ± 0.49^$$$^	0.096
Cholesterol (mg/day)	363.43 ± 142.46	157.94 ± 18.44	206.68 ± 140.82^*$$$*^	424.43 ± 162.31	0.053	275.75 ± 46.51	**<0.001**	241.21 ± 105.00	0.196
Fiber (g/day)	20.07 ± 8.77	27.57 ± 1.33	18.75 ± 8.50^*$$$*^	21.66 ± 9.69	0.400	22.84 ± 3.48	**<0.001**	20.61 ± 9.01	0.316
Glycemic Index (U)	579.55 ± 179.16	487.29 ± 12.21	367.48 ± 131.06^$$$^	685.13 ± 226.21	**0.013**	332.01 ± 38.04	**<0.001**	326.70 ± 121.11	0.131
Glycemic Load (U)	105.21 ± 43.52	63.77 ± 5.47	70.24 ± 35.70	117.32 ± 43.92	0.178	43.37 ± 5.04	**<0.001**	52.03 ± 26.40^$^	**0.008**
TAC (mmol/day)	7.36 ± 3.66	10.85 ± 0.36	8.88 ± 2.72^$$$^	8.22 ± 4.41	0.302	17.09 ± 0.62	**<0.001**	13.90 ± 5.05^$$$^	**<0.001**
HEI (points)	60.28 ± 11.92	86.21 ± 0.99	70.90 ± 12.75^$$$^	56.05 ± 11.11	0.075	91.39 ± 1.80	**<0.001**	74.41 ± 10.07^$$$^	0.836
Meal Frequency (meals/day)	4.65 ± 0.59	3-5	4.59 ± 0.67	5.30 ± 1.25	**0.001**	7	N.A	6.51 ± 1.06	**<0.001**

*CHO*, Carbohydrates; *PQ*, Protein Quality; *SFA*, Saturated Fatty Acids; *MUFA*, Mono Unsaturated Fatty Acids; *PUFA*, Poly Unsaturated Fatty Acids; *n-3 FA*, n-3 Fatty Acids; *TAC*, Total Antioxidant Capacity; *HEI*, Healthy Eating Index; *NA*, Not Applicable.

*p* < 0.05 was set-up as statistically significant. *p*^a^ Differences between Control and RESMENA groups intake before starting the intervention. *p*^b^ Differences between Control and RESMENA scheduled diets. *p*^c^ Differences between Control and RESMENA groups intake at the end of the intervention. $ Differences between the scheduled diets and the real intake at final day in both Control and Resmena groups. ^$^*p* < 0.05; ^$$^*p* < 0.01; ^$$$^*p* < 0.001.

Participants were provided a 7-day menu plan in the RESMENA group and a food exchange system plan in the Control group, as previously described [2]. Usual diet was assessed with a semiquantitative 136-item food frequency questionnaire previously validated in Spain for energy and nutrient intake [14]. A 48-hour weighed food record was required at the beginning and at the end of the study. Diet composition was analyzed using the DIAL software (Alce Ingenieria, Madrid, Spain). The sum of eicosapentaenoic fatty acid and docosahexaenoic fatty acid (EPA + DHA) obtained by the DIAL program was used to estimate n-3 fatty acids consumption. The Healthy Eating Index (HEI) was calculated using also the DIAL software as described elsewhere [23]. The program gives different values between 0 and 100 considering the servings per day of cereals, vegetables, fruits, dairy products and meat. It also takes into account the percentage of energy provided by total and saturated fats, the amount of cholesterol and sodium per day and the variety of the diet. The final score was classified in five categories: > 80 points indicates “excellent diet”; 71–80 points = “very good diet”; 61–70 points = “good diet”; 51–60 = “acceptable diet” and 0–50 points = “inadequate diet”. Protein Quality (PQ) was defined as the ratio of essential amino acid to total dietary protein [24]. Dietary TAC was calculated using the list from Carlsen et al. 2010, which takes into consideration raw or cooked food preparations [25]. They provide a list of the total antioxidant content (mmol/100 g) of more than 3100 foods, beverages, spices, herbs and supplements used worldwide. The TAC value corresponding to the different scheduled/ingested servings per day was calculated. GI and GL were obtained from Foster-Powell et al. [26] (University of Sydney updated website database 2012).

Participants were asked to maintain their normal physical activity during the study, estimated with a 24-h recall at the beginning and the end of the intervention.

### Anthropometric and biochemical assessments

Anthropometric measurements (body weight, height, waist and hip circumferences) were carried out with the subjects in their underwear. Body weight was measured to the nearest 0.1 kg using a Tanita SC-330, (Tanita corp, Japan). Height was estimated with a stadiometer (Seca 713 model, Postfach, Germany) to the nearest 1 mm. BMI was calculated as the body weight divided by the squared height (kg/m^2^). Waist and hip circumferences were measured with a commercial measure tap following validated protocols [19]. Total body fat was measured by a bioelectric impedance Tanita SC-330 (Tanita corp, Japan) and by DXA (Lunar Prodigy, software version 6.0, Madison, WI) as described elsewhere [19].

Glucose, total cholesterol, high density lipoprotein-cholesterol (HDL-c), triglycerides, alanine aminotransferase (ALT) and aspartate aminotransferase (AST) serum concentrations were measured in an autoanalyser Pentra C-200 (HORIBA ABX, Madrid, Spain) with specific kits. Insulin concentrations were determined by an enzyme-linked immunosorbent assay kit (Mercodia, Uppsala, Sweden) in a Triturus autoanalyzer (Grifols SA, Barcelona, Spain). Insulin resistance was estimated by the HOMA index {HOMA-IR = [glucose (mmol/L) × insulin (μU/ml)]/22,5} [5]. Low-density lipoprotein-cholesterol (LDL-c) levels were calculated following the Friedewald formula: LDL-c = TC-HDL-c - VLDL [10].

### Statistical analyses

The results are expressed as mean ± SD. Normality distributions of the measured variables were determined according to the Shapiro–Wilk test. Differences between the beginning and the end of the period were analyzed by a paired *t*-test. Only those completely the study were analysed. The analysis between both diets (RESMENA *vs.* Control) was performed through an independent measures *t*-test. A linear regression analysis was applied to assess the potential relationships and associations among the different components of the diet and anthropometrical and biochemical parameters variation. Chi-square test was carried out to compare the percentage of participants from Control and RESMENA dietary groups included in the high-TAC group. A two-way ANOVA was performed in order to assess diet and sex interactions. Analyses were carried out using SPSS 15.1 software for Windows (SPSS Inc, Chicago, USA). Values of p < 0.05 were considered as statistically significant.

## Results

### Food intake assessment

There were available data about food intake of 90 participants (48 from Control group and 42 from RESMENA group). No differences were found in food energy intake between the experimental groups at the study baseline, except for total fat and saturated fat intake, GI and meal frequency (Table 2).

The RESMENA group reported a significantly higher protein and lower carbohydrate intake and the protein/carbohydrate ratio was also higher in this group at the end of the study (Table 2). There were no significant differences between groups either for PQ or total fat intake, but significant differences were found regarding fatty acids profile (Table 2). There were no significant differences in cholesterol intake after the intervention. Concurrently, no differences were found in fiber intake, neither in GI or HEI score. GL was significantly lower (*p* = 0.008) and TAC significantly higher (*p* < 0.001) in RESMENA group, with respect to the AHA group. As designed, consuming the RESMENA diet had an average intake (6.5 meals/day) significantly higher than the control one (4.5 meals/day).

The analysis of 48-h food records showed that in both groups the energy intake was in accordance with the prescribed patterns (Table 2). Protein, saturated fatty acid, polyunsaturated fatty acid (PUFA) and n-3 fatty acids intakes, as well as GL and meal frequency of the Control diet were in agreement with the scheduled pattern (Table 2). RESMENA group adjusted to PUFA, cholesterol and fiber intake and also the GI and meal frequency. RESMENA group did not reach completely the expected values of some components although it achieved an improvement comparing to baseline values.

### Anthropometric and biochemical parameters

Energy restriction resulted in a mean body weight loss of 6.73 ± 0.71 kg in the Control diet and 7.09 ± 0.82 kg in the RESMENA diet, with no statistical differences between groups (Table 3). Consequently, BMI was significantly lowered in both groups. Every anthropometrical parameter was significantly reduced after the slimming treatments, with no differences between both dietary groups (Table 3). Control and RESMENA groups significantly reduced total cholesterol and triglycerides but there was no significant reduction in LDL-c. Only the AHA group had a significant decrease of the HDL-c values (*p* = 0.001). No changes were observed in the atherogenic ratios TC:HDL-c and LDL-c:HDL-c in none of the groups. Both groups significantly improved glucose metabolism (Table 3). The Control diet, but not the RESMENA one induced a significant reduction in ALT (*p* < 0.001 *vs. p* = 0.936) and AST (*p* = 0.001 *vs. p* = 0.740) transaminases levels, obtaining significant differences among groups (Table 3). No differences between groups were found in any of the other biochemical parameters (Table 3).

**Table 3:** Changes in selected parameters in Control and RESMENA groups after 8 weeks of nutritional intervention

**Variable**	**Control group (n = 48)**			**Resmena group (n = 48)**			
	**Visit 1 (day 0)**	**Visit 2 (day 60)**	***p***^***a***^	**Visit 1 (day 0)**	**Visit 2 (day 60)**	***p***^***b***^	***p***^**c**^
Weight (kg)	99.45 ± 19.21	92.72 ± 18.50	**<0.001**	100.00 ± 16.58	92.91 ± 15.76	**<0.001**	0.555
BMI (kg/m^2^)	36.15 ± 4.95	33.70 ± 4.80	**<0.001**	35.55 ± 4.40	33.03 ± 4.24	**<0.001**	0.732
Waist circumference (cm)	110.94 ± 13.41	104.18 ± 12.29	**<0.001**	111.25 ± 12.30	103.78 ± 11.66	**<0.001**	0.432
Waist/hip ratio	0.96 ± 0.10	0.94 ± 0.09	**<0.001**	0.96 ± 0.10	0.93 ± 0.10	**<0.001**	0.355
Bioimpedance Fat mass (kg)	38.97 ± 10.87	33.68 ± 10.22	**<0.001**	39.23 ± 9.50	33.84 ± 9.09	**<0.001**	0.886
DXA Fat mass (kg)	42.13 ± 10.18	37.50 ± 10.39	**<0.001**	42.56 ± 9.18	37.30 ± 8.95	**<0.001**	0.208
TC (mg/dl)	221 ± 39	204 ± 39	**0.001**	219 ± 48	203 ± 46	**0.020**	0.943
HDL-c (mg/dl)	46 ± 10	42 ± 9	**0.001**	43 ± 10	41 ± 10	0.050	0.158
LDL-c (mg/dl)	140 ± 36	133 ± 35	0.085	137 ± 41	131 ± 40	0.374	0.888
TC/HDL-c ratio	4.94 ± 1.02	4.96 ± 0.94	0.856	5.18 ± 1.24	5.04 ± 1.24	0.398	0.419
LDL-c/HDL-c ratio	3.09 ± 0.77	3.22 ± 0.78	0.178	3.20 ± 0.83	3.23 ± 0.92	0.803	0.623
TG (mg/dl)	176 ± 10	145 ± 70	**0.005**	194 ± 123	151 ± 99	**<0.001**	0.421
Glucose (mg/dl)	121.02 ± 33.87	107.98 ± 13.71	**0.006**	123.81 ± 37.82	110.22 ± 26.18	**0.016**	0.939
Insulin (IU/mg)	15.30 ± 11.46	9.32 ± 7.18	**<0.001**	14.36 ± 8.30	9.14 ± 6.13	**<0.001**	0.557
HOMA index	4.69 ± 3.77	2.61 ± 2.31	**<0.001**	4.48 ± 3.01	2.60 ± 2.00	**<0.001**	0.686
ALT (U/L)	37.60 ± 21.04	27.03 ± 10.70	**<0.001**	29.13 ± 11.59	29.28 ± 14.20	0.936	**0.001**
AST (U/L)	25.81 ± 10.84	20.68 ± 6.18	**0.001**	21.90 ± 6.01	22.20 ± 6.14	0.740	**0.002**

*BMI*, Body Mass Index; *DXA*, Dual-energy X-ray Absorptiometry; *HDL-c*, High Density Lipoprotein-cholesterol; *LDL-c*, Low Density Lipoprotein-cholesterol; *TG*, Triglycerides; *HOMA*, Homeostasis Model Assessment; *ALT*, Alanine aminotransferase; *AST*, Aspartate aminotranferase. *p* < 0.05 was set-up as statistically significant.

*p*^*a*^ differences between baseline and final values in Control group*. p*^***b***^ differences between baseline and final values in RESMENA group. *p*^c^ differences between changes in Control group as compared with RESMENA group at the end of the intervention.

**Figure 2:**
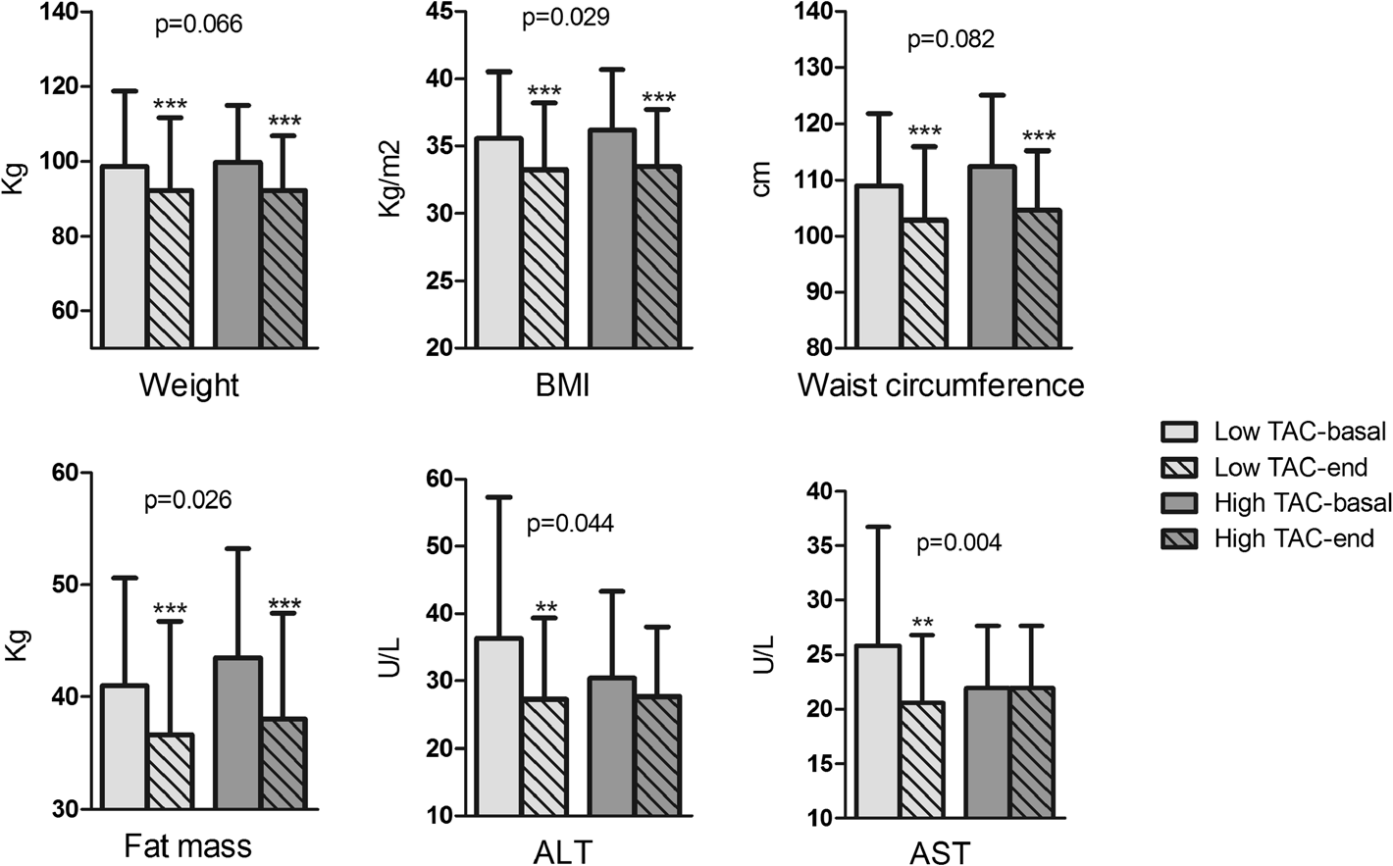
**Changes on selected parameters categorized by low (<10.629 mmol day^-1^, n = 45) or high (>10.629 mmol day^-1^, n = 45) Total Antioxidant Capacity.** BMI: Body Mass Index; ALT: Alanine aminotransferase; AST: Aspartate aminotranferase. p-values comparing the differences between low-TAC and high-TAC groups.

Dietary TAC was the major influential factor, as body weight (*p* = 0.034), BMI (*p* = 0.026), fat mass (*p* = 0.015) and AST (*p* = 0.004) were significantly improved by this variable (Table 4). Dietary TAC also seemed to have a potential effect in ALT variations (*p* = 0.062). Concerning GI/GL, a trend towards an influence of insulin was found (Table 4). Since dietary TAC seemed to be the most influencing variable among the dietary analyzed elements, we categorized the sample considering the median value in: high- TAC (> 10.629 mmol day^-1^) or low- TAC (<10.629 mmol day^-1^) (Figure 2). As expected, the percentage of subjects from the RESMENA group (71%) included in the high-TAC group was significantly higher (p < 0.001) as compared with the Control group (29%). Body weight losses were marginally (*p* = 0.066) different when the subjects were categorized by the dietary TAC (Figure 2). Thus, the group with a higher dietary TAC showed a greater body weight reduction (−7.52 ± 0.64 kg) when compared with the low-TAC group (−6.35 ± 0.86 kg). Waist circumference decreased marginally towards significant differences between the two groups (*p* = 0.082) being greater in the high-TAC one (−7.77 ± 2.07 cm *vs.* -6.15 ± 0.31 cm). Fat mass was significantly reduced in both TAC groups and differences between them regarding densitometry fat mass kilograms (*p* = 0.026) were found. The low-TAC group significantly reduces ALT (*p* = 0.001) and AST (*p* = 0.002) transaminases levels, being statistically significant regarding the high-TAC group (*p* = 0.044 and *p* = 0.004, respectively) (Figure 2).

**Table 4:** Regression analysis, with change in phenotypical measurements as dependent variable and dietary components as the independent

	**Meal Frequency**		**Protein Intake**		**n-3 FA intake**		**TAC**		**GI**		**GL**		**HEI**		**Model p**	**Corrected r**^**2**^
**Variable**	**B**	**p**	**B**	**p**	**B**	**p**	**B**	**p**	**B**	**p**	**B**	**p**	**B**	**p**		
Weight (Kg)	0.899	0.146	0.806	0.206	0.252	0.681	1.274	**0.034**	0.085	0.889	−0.519	0.399	1.140	0.059	**0.024**	0.122
BMI (kg/m^2^)	0.275	0.215	0.218	0.342	0.032	0.884	0.480	**0.026**	−0.025	0.910	−0.194	0.378	0.430	**0.047**	0.132	0.060
Waist circumference (cm)	0.731	0.446	1.392	0.157	−0.107	0.910	1.618	0.083	0.064	0.946	−0.722	0.447	0.624	0.508	0.559	−0.014
Bioimpedance																
Fat mass (kg)	0.613	0.394	−0.036	0.962	1.358	0.053	1.185	0.091	−0.215	0.760	−0.424	0.552	0.639	0.365	0.128	0.061
DXA																
Fat mass (kg)	0.949	0.063	0.819	0.120	0.434	0.392	1.211	**0.015**	0.050	0.921	0.154	0.763	0.806	0.109	0.133	0.059
Total Cholesterol (mg/dl)	3.886	0.670	0.444	0.962	−13.795	0.123	5.041	0.573	−2.462	0.783	−8.968	0.319	−16.091	0.071	0.381	0.010
HDL-c (mg/dl)	−0.786	0.612	−2.045	0.199	−2.055	0.178	0.558	0.714	−0.951	0.531	−1.558	0.308	−0.247	0.872	0.311	0.021
LDL-c (mg/dl)	5.174	0.522	1.930	0.817	−13.268	0.095	−0.164	0.984	−2.581	0.745	−4.266	0.594	−13.979	0.077	0.348	0.015
TG (mg/dl)	−2.511	0.865	2.792	0.855	7.639	0.601	23.238	0.107	5.349	0.712	−15.720	0.281	−9.324	0.522	0.471	−0.003
Glucose (mg/dl)	3.560	0.648	−8.493	0.290	6.908	0.370	−5.614	0.464	2.408	0.753	7.928	0.303	9.467	0217	0.781	−0.035
Insulin (IU/mg)	−1.662	0.233	−1.144	0.427	−1.814	0.188	−0.664	0.629	−2.546	0.061	−2.572	0.060	−1.597	0.246	0.198	−0.063
HOMA	−0.539	0.299	−0.487	0.364	−0.151	0.770	−0.371	0.468	−0.666	0.190	−0.465	0.365	−0.382	0.457	0.894	0.044
ALT (U/L)	−6.226	**0.049**	−6.852	**0.035**	−4.413	0.160	−5.795	0.062	3.721	0.232	3.708	0.238	5.855	0.060	0.066	0.089
AST (U/L)	−3.557	0.051	−4.539	**0.015**	0.084	0.963	−5.046	**0.004**	1.610	0.371	1.580	0.384	4.699	**0.008**	**0.020**	0.131

*n-3 FA*, n-3 Fatty Acids; *TAC*, Total Antioxidant Capacity; *GI*, Glycemic Index: *GL*, Glycemic Load; *HEI*, Healthy Eating Index; *BMI*, Body Mass Index; *DXA*, Dual-energy X-ray Absorptiometry; *HDL-c*, High Density Lipoprotein-cholesterol; *LDL-c*, Low Density Lipoprotein-cholesterol; *TG*, Triglycerides; *HOMA*, Homeostasis Model Assessment; *ALT*, Alanine minotransferase; *AST*, Aspartate aminotranferase. *p* < 0.05 was set-up as statistically significant.

**Figure 3:**
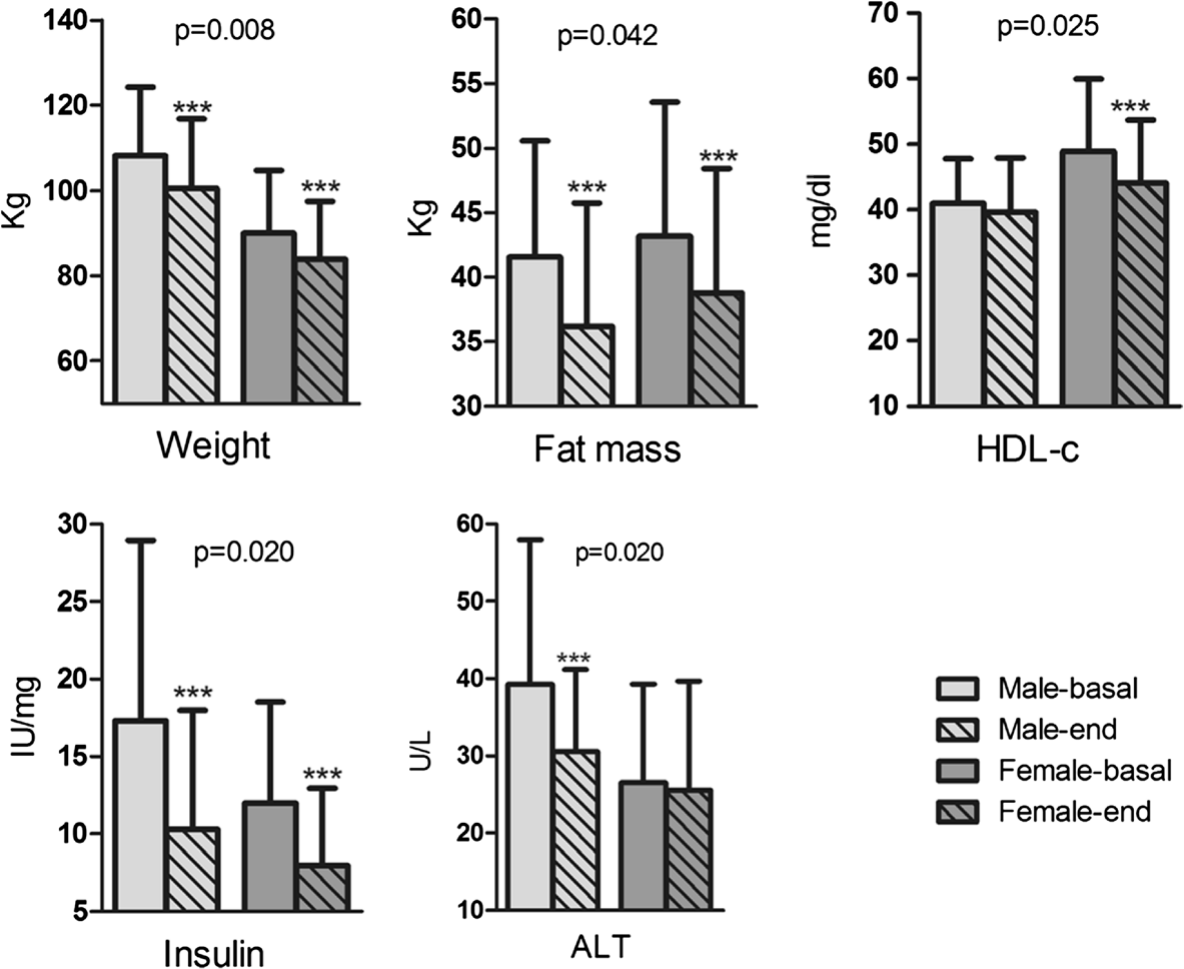
**Changes on anthropometric and biochemical selected parameters regarding gender, male (n = 51) or female (n = 45).** HDL-c: High Density Lipoprotein-cholesterol; ALT: Alanine aminotransferase. *p* -values comparing the differences between male and female groups.

As expected, gender variation influenced anthropometrical and biochemical parameters changes (Table 5). Body weight loss was significantly higher in males than in females (*p* = 0.008), as well as fat mass reduction (*p* = 0.042) (Figure 3). Interestingly, men showed a statistically significant decrease of insulin blood levels (*p* = 0.020). Concurrently, ALT values were significantly reduced in men, while we did not found any changes in this marker in females group (Figure 3). Regarding dietary TAC influence, differences concerning gender were found. Women with higher TAC levels, showed a significantly greater reduction of body weight (*p* = 0.019), BMI (*p* = 0.028) and fat mass (*p* = 0.007), while there were not any variable differences between high or low TAC in the male group.

**Table 5:** Analysis assessing diet and sex interactions concerning anthropometric and biochemical markers

**Variable**	**Groups**				**ANOVA 2×2**		
	**Control-men (n = 27)**	**Control-women (n = 21)**	**RESMENA-men (n = 24)**	**RESMENA-women (n = 24)**	**DIET**	**SEX**	Diet* sex
ΔWeight (Kg)	−7.60 ± 3.29	−5.61 ± 2.49	−7.73 ± 2.58	−6.45 ± 3.09	0.416	**0.007**	0.550
ΔBMI (kg/m^2^)	−2.56 ± 1.12	−2.29 ± 1.03	−2.55 ± 0.90	−2.49 ± 1.10	0.677	0.452	0.620
ΔWaist circumference (cm)	−6.57 ± 4.02	−7.01 ± 5.87	−8.15 ± 2.88	−6.80 ± 4.65	0.454	0.620	0.324
ΔBioimpedance Fat mass (kg)	−6.08 ± 4.92	−4.28 ± 1.84	−5.90 ± 2.64	−4.89 ± 3.50	0.764	0.054	0.582
ΔDXA Fat mass (kg)	−5.13 ± 2.80	−3.99 ± 1.49	−5.71 ± 1.95	−4.78 ± 2.75	0.156	**0.034**	0.835
ΔTotal Cholesterol (mg/dl)	−15.31 ± 35.58	−19.05 ± 30.86	−22.25 ± 44.19	−10.42 ± 49.55	0.921	0.635	0.362
ΔHDL-c (mg/dl)	−2.01 ± 7.10	−6.67 ± 6.98	−0.61 ± 7.29	−3.32 ± 6.03	0.098	**0.011**	0.495
ΔLDL-c (mg/dl)	−6.12 ± 25.04	−7.48 ± 27.41	−10.82 ± 40.17	−0.51 ± 47.30	0.880	0.553	0.440
ΔTG (mg/dl)	−35.88 ± 78.11	−24.50 ± 61.90	−54.08 ± 88.48	−32.96 ± 69.79	0.399	0.304	0.757
ΔGlucose (mg/dl)	−18.53 ± 36.01	−5.91 ± 19.86	−7.33 ± 26.55	−19.84 ± 46.17	0.847	0.994	0.079
ΔInsulin (IU/mg)	−7.68 ± 9.02	−3.37 ± 3.58	−6.26 ± 5.02	−4.16 ± 5.25	0.690	**0.022**	0.486
ΔHOMA	−2.64 ± 2.65	−1.35 ± 1.68	−1.74 ± 1.63	−2.02 ± 2.83	0.818	0.293	0.099
ΔALT (U/L)	−13.68 ± 19.36	−6.52 ± 12.77	−3.33 ± 10.70	3.64 ± 15.38	**0.001**	**0.026**	0.975
ΔAST (U/L)	−6.49 ± 11.74	−3.37 ± 6.16	−0.28 ± 6.13	0.88 ± 6.51	**0.003**	0.210	0.562

*BMI*, Body Mass Index; *DXA*, Dual-energy X-ray Absorptiometry; *HDL-c*, High Density Lipoprotein-cholesterol; *LDL-c*, Low Density Lipoprotein-cholesterol; *TG*, Triglycerides; *HOMA*, Homeostasis Model Assessment; *ALT*, Alanine aminotransferase; *AST*, Aspartate aminotranferase. p < 0.05 was set-up as statistically significant.

## Discussion

This study compared the effects of a novel dietary strategy with the AHA pattern [20], considered to be effective to counteract obesity. To our knowledge, this is a pioneer intervention study in patients with MetS features evaluating the effects of an energy restricted diet based on food selection, including a modified macronutrient distribution, an increase in meal frequency, as well as the presence of bioactive ingredients, such as fiber and n-3 fatty acids, and controlling the GI/GL, dietary TAC and HEI score [19].

Evaluating the two prescribed dietary strategies as a whole, both were for weight loss and improved anthropometric and biochemical markers, with minor differences between them. Waist circumference was reduced in all the participants. However, when considering the IDF criteria for abdominal obesity (> 90 cm. in men and > 80 cm. in women) 4 people reached lower values after the dietary intervention (1 man and 1 woman from the Control group, 1 man and 1 woman from the RESMENA group).

Specifically, the individual role of each diet component was analyzed in order to assess the dietary components with major influence on these measurements in a MetS sample. In this sense, several works have studied dietary components effects separately in humans, but available results are controversial [5,7,13].

Recent investigations have focused on the role of the macronutrient distribution in weight reduction treatments instead of considering only calorie restriction [27]. In this context, increasing the dietary protein content has been a frequently-used strategy, due to the increased satiety with a reduction of energy intake in subsequent meals and the higher thermogenic effect attributed to them [6,27]. Thus, in the current work it was prescribed a dietary pattern including the 30% of total caloric value as protein at the expense of carbohydrates (40% total caloric value). This profile did not apparently induce changes on any anthropometrical parameters between the experimental groups. Different studies have shown high-protein intake effects in relation with body weight changes, specifically on weight regain. However, those effects were found in the long-term [10] while the present work focused on the effects of an 8-week dietary treatment.

Interestingly, our study showed an inverse association between protein intake and both ALT and AST transaminases levels. These enzymes are unspecific markers of hepatic function [28]. Low-serum transaminases levels are found under normal conditions, indicating proper function of the liver, while increased serum values are related to hepatic dysfunction [28]. They have shown a correlation with insulin resistance and later development of diabetes, liver lipid content and features of non-alcoholic fatty liver disease. The experimental data are consistent with those other studies and suggest that moderately-high protein diets could influence negatively liver function. Concerning transaminases modification, it is important to notice that some differences were found before starting the intervention. For that reason we performed a percentage of change analyses in addition to the absolute values approach, in order to attenuate the possible bias. However, similar outcomes were obtained when applying this analysis, therefore, absolute values were maintained in Table 3.

Regarding the increased meal frequency, no differences in body weight loss in the context of iso-energetic energy-restricted diets were found, as also was reported by Cameron et al. 2009 [7]. Nevertheless, a direct relationship between increased meal frequency and the loss of fat mass was observed. With respect to biochemical parameters, no influences were found. However, Heden et al. 2012 observed that meal frequency differentially altered triglycerides and insulin postprandial concentrations [29].

The beneficial properties of n-3 fatty acids have been widely studied [9,16]. In our study, a positive relationship between n-3 fatty acids intake and the reduction of fat mass was detected, being consistent with previous human studies [16].

GI and GL are two concepts that refer to the absorption rate of carbohydrates [26]. Increased values have been reported as potential type-2 Diabetes Mellitus risk factors [5]. In this sense, an encouraging result was obtained in our study, since we found a trend towards a reduction in insulin levels related to lower values of GI and GL in diet, in agreement with Bao et al. 2010 [30].

In order to assess the quality of the diet, numerous authors have designed and developed indexes or scores such as the Healthy Eating Index, the Alternate Healthy Eating Index or the Diet Quality Index and derivatives [14]. Most of them, take into consideration the Mediterranean Diet guidelines, widely recognized as a healthy pattern [30]. They consist on a single score that results from computing different component such as foods, food groups or a combination of foods and nutrients. In this context the HEI score was selected, obtained from the DIAL software. It takes into account macro and micronutrients intake, as well as food variety, to design the RESMENA diet. Considering the HEI score, a trend towards influencing weight loss and total cholesterol, LDL-c, ALT and AST levels was found. Other works evaluating Mediterranean-based patterns reported similar results regarding lipid metabolism and hepatic function markers [31,32].

The most relevant finding of this study is in relation to dietary TAC. This parameter has been recently considered as a useful tool to assess the effects of dietary antioxidants, since it has been positively associated with plasma total antioxidant capacity [33]. After 8 weeks of intervention, we evidenced positive associations between the dietary TAC and the reduction of weight, BMI, waist circumference and fat mass. Regarding these anthropometric measurements, our findings are in accordance with previous studies that also reported benefits of dietary TAC and antioxidants compounds on adiposity and obesity indicators [31,34]. This link may be associated with a reduction of cardiovascular risk, as previously described in other populations [35]. We also found an effect on ALT and AST transaminases suggesting an impact of the dietary TAC on hepatic metabolism. These data suggest that dietary TAC, as a measure of antioxidant intake, must be useful to assess the role of antioxidant intake as a single factor in the field of antioxidant consumption and disease prevention or therapy.

Contrary to most of the evaluated parameters, HDL-c values did not improve with none of the dietary treatments, which can be explained by the fact that the reduction on total cholesterol entails a reduction of this cholesterol fraction too, as previously reported by other researchers [36]. However, some other authors found higher HDL-c serum levels after similar dietary interventions [37] especially when containing fish or fish derived products [16,38]. Despite our dietary strategy also focused on n-3 fatty acids intake, the participants did not reach a perfect adherence to this point, so that this can also contribute to the fact that HDL-c was found to decrease after the dietary intervention. Other important factor in relation to HDL-c is physical activity. In this sense, the participants were asked to maintain the normal activity and no exercise advice was given, which may allow discard differences due to physical activity changes.

Differences between males and females regarding anthropometric and biochemical parameters have been widely investigated [1,7,39]. In this sense, we analyzed gender influence on the studied variable changes. Body weight loss was higher in men than women, due to the also higher restriction regarding absolute amount of calories. This greater reduction of weight was accompanied by an also higher decrease of fat mass, as previously reported [7]. Regarding biochemical parameters, insulin as well as ALT blood levels were significantly decreased within this group in accordance with previous studies [1]. On the contrary, HDL-c reduction was greater among the women group, as other studies reported [40], which confirms the influence of sex on this cholesterol fraction. Taken together, these outcomes indicate that gender may be taken into account in order to design specific dietary plans for males and females.

This work could benefit of increasing sample size. Additionally, although the adherence to the diet was acceptable, this kind of treatments could show higher benefits when reaching a stricter follow-up of the dietary pattern. On the other hand, we have analyzed the effects of this treatment on obese adults with MetS features. The effectiveness of this pattern should be also evaluated in a younger population since obesity and MetS rates have increased alarmingly among childhood [41] and they represent a development problem [42] leading to an increased morbimortality at the adult age [43].

## Conclusion

Taken together, the results of this study indicate that RESMENA diet could be considered as a new dietary strategy in order to improve anthropometrical and biochemical parameters in obese subjects presenting MetS manifestations. Furthermore, dietary TAC seems to be, among all the analyzed dietary aspects, the most relevant one in the obesity related markers.

## Abbreviations

AHA: American Heart Association; ALT: Alanine aminotransferase; AST: Aspartate aminotransferase; BMI: Body mass index; DXA: Dual-energy X-ray Absorptiometry; GI: Glycemic index; GL: Glycemic load; HDL-c: High Density Lipoprotein-cholesterol; HEI: Healthy Eating Index; LDL-c: Low Density Lipoprotein-cholesterol; MetS: Metabolic syndrome; PQ: Protein Quality; PUFA: Poly Unsaturated Fatty Acids; TAC: Total antioxidant capacity; WHO: World Health Organisation.

## Competing interests

The authors declare that they have no competing interests.

## Authors’ contributions

The authors contributions were as follows: PLL contributed to the design and the fieldwork, data collection, analysis and writing of the manuscript. RI and IA were involved in the design and the fieldwork. IBP contributed to the sample collection, interpretation and critical reading of the last version. SNC and LF were involved in recruitment and volunteers selection as well as in critical reading of the manuscript. MAZ was responsible for the general coordination, follow-up, design and financial management. JAM, project co-leader, was responsible of the follow-up, design, financial management and editing of the manuscript. All the authors actively participated in the manuscript preparation, as well as read and approved the final manuscript.
